# Demodulation Method of F-P Sensor Based on Wavelet Transform and Polarization Low Coherence Interferometry

**DOI:** 10.3390/s20154249

**Published:** 2020-07-30

**Authors:** Jiwen Cui, Yizhao Niu, Hong Dang, Kunpeng Feng, Xun Sun, Jiubin Tan

**Affiliations:** 1 Center of Ultra-precision Optoelectronic Instrument, Harbin Institute of Technology, Harbin 150080, China; nyz_hit@163.com (Y.N.); danghong@hit.edu.cn (H.D.); sx2503962673@163.com (X.S.); jbtan@hit.edu.cn (J.T.); 2 Key Lab of Ultra-precision Intelligent Instrumentation (Harbin Institute of Technology), Ministry of Industry and Information Technology, Harbin 150080, China; 3 Institute of Optical Communication Engineering, College of Engineering and Applied Sciences, Nanjing University, Nanjing 210093, China; kpfeng@nju.edu.cn; 4 Key Laboratory of Intelligent Optical Sensing and Manipulation, Ministry of Education, Nanjing 210093, China

**Keywords:** low-coherence interference (LCI), dispersion, extremum locations, wavelet tool

## Abstract

Polarized low-coherence interferometry (PLCI) is widely used for the demodulation of Fabry–Perot (F-P) sensors. To avoid the influence of noise and dispersion on interference fringes, this paper proposes a data processing method in which the wavelet tools are applied to extract useful information from the extremum locations and envelope center of the fringes. Firstly, the wavelet threshold denoising (WTD) algorithm is used to remove electrical noise, and the complex Morlet wavelet is used to extract the fringe envelope. Based on this, the envelope center is used to predict the extremum locations of the specified order in its adjacent interval, the predicted locations are used as references to track the exact extremum locations, and the middle location of the peak and valley values is obtained to demodulate the F-P cavity accurately. The validity of this demodulation theory is verified by an air F-P cavity whose cavity length varies from 17 to 20 μm. With a sampling interval of 30 nm, the experimental results indicate that the repeatability accuracy is higher than 6.04 nm, and the resolution is better than 4.0 nm.

## 1. Introduction

Benefit from their electromagnetic interference immunity, small size, low loss, and long-term stability, fiber Fabry–Perot (F-P) sensors have been widely used to measure displacement, temperature, and air pressure, etc. [1,2,3,4,5] In these applications, the sensing information is implicit in the interferometric spectra of the F-P sensors [6]. Therefore, advances in spectral demodulation methods with high resolution and accuracy are conducive to the improvement of the system performance. In 1999, May et al. [7] reported a self-calibration interference/intensity (SCIIB) configuration, wherein the output of the F-P sensor was processed by an optical filter. This method eliminated the accuracy degeneration induced by the fluctuations of the light source power, but the range of this method is limited by the free spectral range of the interferometer, as well as the nonlinear error [8]. After that, Xu [9] proposed in 2005, a wide-range configuration by employing an optical spectrum analyzer to collect the fringes. This method is relatively simple, but it is often puzzled by the inherent defects of the optical spectrum analyzer, e.g., slow dynamic response speed, high cost, and difficulties of reuse. In recent years, due to the advantages of no range limitation, absolute parameter measurement, and easily reuse, the low-coherence interference (LCI) demodulation method has been applied to optical fiber F-P sensing [10,11,12]. The output optical signal passes through the LCI optical path, wherein the optical wedge can produce continuously variable OPDs, and the fringe distribution formed by the cross-correlation reflects the F-P cavity information. Belleville et al. [13] used a Fizeau interferometer consisting of an air wedge to demodulate the F-P sensors. However, the synchronous demodulation of multi-cavities demands a wedge angle less than 0.1°, the difficulties in manufacturing such a wedge may introduce extra errors to the sensing system. To solve this problem, Wang et al. [14] replaced the air wedges with a birefringent crystal optical wedges (BWs), and a fixed wedge angle of 5° was achieved. Nevertheless, due to the dispersion of BWs [15,16], the Gaussian distribution of the light emitted from the fiber, and the fluctuation of the light source, the fringes are distorted. Shuang et al. [17] modified the models of BWs by taking dispersion into account. Based on that model, Wang et al. further proposed a phase-based method in which the demodulation is performed by the maximum value location of the zero-order fringe, and the orders of the fringes were evaluated by envelope center. However, due to the amplitude distortion of the fringes, centroid method and Fourier transform method [18] may introduce significant deviations to the determination of envelope center, which limits the accuracy and practicality of these demodulation methods. Sandoz [19] used wavelet transform to process white light interference fringes as early as 1997, decoded fringe envelope by constructing Gaussian envelope mother wavelet, and demodulated according to phase shift information. Cherbliez et al. [20] proposed a real-time white light interference analysis method based on complex Morlet wavelet. The phase of the signal can be calculated by using the phase characteristics of the wavelet so that the displacement of the object can be measured directly. Hongjie et al. [21] use the complex Morlet wavelet as the mother wavelet to extract the envelope of the white light interference signal. However, the phase information directly obtained by wavelet processing is sensitive to the amplitude distortion.

**Figure 1:**
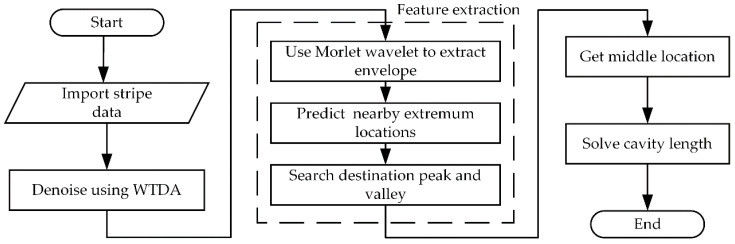
Fabry–Perot (F-P) cavity demodulation flowchart.

This paper proposes a wavelet-based algorithm demodulating the sensing information from both envelope center and extremum locations. As shown in Figure 1, the core of the method is how to calculate the envelope center and the extremum locations of target fringes. To overcome the influence of the amplitude distortions, this paper firstly applies WTD tools to extract interferometric fringes and then evaluate the location of the envelope center through complex Morlet wavelet envelope extraction (MWEE). After that, the nearest extremum locations to the evaluated envelope center are targeted and traced, and the sensing information can be derived from the variations of the target extremum locations. The effects of wavelet basis and decomposition layer numbers on the results of denoising and envelope extraction are discussed and the differences between Fourier transform and complex Morlet wavelet in envelope extraction are compared. Finally, the validity of the proposed method is verified by monitoring the cavity length of the F-P sensor, the data processing results show that the fringe demodulation scheme is immune from amplitude distortions, dispersion, etc., and thus can be utilized in various applications.

This paper is structured as follows: Section 2 models the patterns of LCI fringes in which the dispersion of birefringent crystal is taken into account, a demodulation scheme based on envelope center extraction and extremum locations trace is then proposed to eliminate the distortion of polarized low-coherence interferometry (PLCI) fringes. Section 3 demonstrates the wavelet tools employed to extract the sensing information of the fringes. Section 4 tests the performances of the demodulation scheme. Section 5 concludes the article.

## 2. Demodulation Principle and Scheme

**Figure 2:**
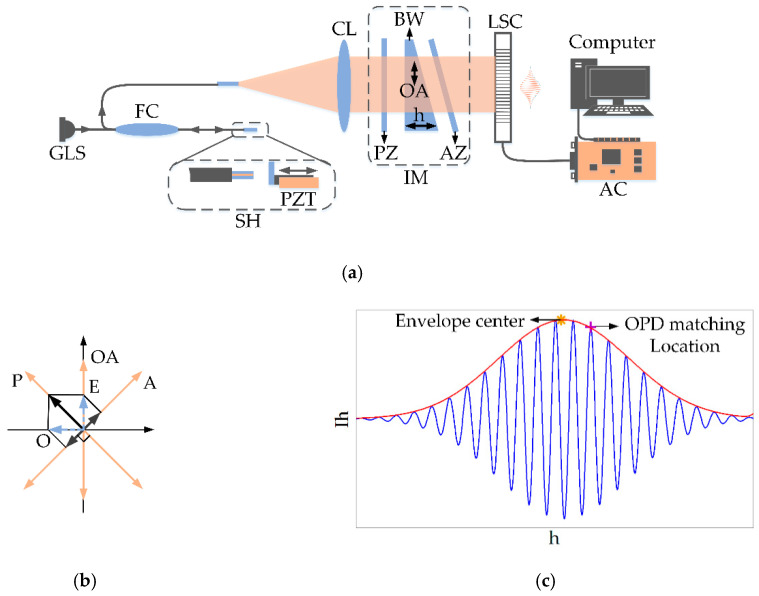
Polarized low-coherence interferometry (PLCI) demodulation system: (**a**) structure diagram; (**b**) geometrical relationship of the components in interference module (IM); (**c**) theoretical distribution of interference fringe.

The schematic diagram of the proposed PLCI demodulation system is shown in Figure 2a. The Gaussian light source (GLS) serves as a PLCI light source illuminating the F-P cavity via the fiber coupler (FC) and the F-P sensor head (SH). With a given cavity length, the F-P cavity could reflect the spectrum with a specific distribution. The reflected beam is then collected by the collimator lens (CL) and decoupled by the interference module (IM) consisting of a polarizer (PZ), BW, analyzer (AZ). Figure 2b demonstrates the geometrical relationship of the components in IM, the polarization direction of AZ is aligned perpendicularly to that of PZ, and the optical axis direction of the birefringent wedge (OA) is on the angular bisector. The PLCI fringe is collected by a line scan camera (LSC), and the data is processed to recover the reflectance spectrum of the F-P cavity.

Assuming that the spectrum of the light source is Gaussian-shaped:

(1) $$I_{i}(k)=I_{c}\frac{2\sqrt{\ln2}}{\sqrt{\pi }\Delta k}\exp\left\{-{\left[\frac{2\sqrt{\ln2}(k-k_{0})}{\Delta k}\right]}^{2}\right\}$$

where, k is the wavenumber, $k_{0}$ is the center wavenumber, and Δk is the full width at half maximum of the spectrum. The reflection spectrum of the F-P sensor is obtained by the principle of multi-beam interference [22]. The relationship between the output spectral form and the cavity satisfies the relationship:

(2) $$I_{r}=I_{i}\frac{4R\sin^{2}\left(\frac{\delta }{2}\right)}{{\left(1-R\right)}^{2}+4R\sin^{2}\left(\frac{\delta }{2}\right)}$$

where, δ is the phase difference introduced by a single transition through the cavity, and it has a relationship with the cavity length d of the F-P cavity δ=2nkd, R is the reflectivity of the lateral wall of the F-P cavity. $I_{r}$ interferes again by the birefringence of the wedge to form a fringe distribution in the direction of thickness change, Δn represents the birefringence of the optical wedge material, and its first-order Maclaurin expansion at $k=k_{0}$ is $\Delta n\approx \Delta n_{0}+\beta \left(k-k_{0}\right)$, $\Delta n_{0}$, and β are the refractive index difference and slope of the optical wedge material at $k_{0}$, respectively. The integrated result of the light intensity at the thickness h is expressed as

(3) $$I_{h}=C_{1}+C_{2}M\left(h\right)\cos\left(\varphi \left(h\right)\right)$$

Among them, M(h) and φ(h) represent the envelope and phase characteristics of fringe wave components respectively. It can be inferred that the expression of a is as follows:

(4) $$M\left(h\right)=\exp\left(\frac{-{\left(\left(\left(\Delta n_{0}+\beta k_{0}\right)h-2d\right)\Delta k\right)}^{2}}{4\times 4\ln2}\right)$$

(5) $$\varphi \left(h\right)=k_{0}\left(\Delta n_{0}h-2d\right)$$

It is clear that the intensity distribution of the PLCI outputs is a Cosine curve modulated by a Gaussian envelope, Figure 2c depicts the simulation results of the fringe. The extremum locations of the cosine curve are at $h_{m}=\left(1/\Delta n_{0}\right)\left(m\pi /k_{0}+2d\right)$, m=1,2,…, and $h_{m}$ contains the locations of the peak and valley; the center of the Gaussian envelope is at $h_{c}=2d/\left(\Delta n_{0}+\beta k_{0}\right)$. The alters of the cavity length can be directly demodulated by tracking either envelope center and extremum locations, but in practice, the power fluctuations of the GLS, the spectral deviations induced by fiber scatterings and the background noise may distort the Gaussian envelope, and thus make the envelope center evaluation inaccurate. In comparison with envelope center, extremum locations are mainly determined by the local variation of the fringes, therefore, the amplitude distortion of the whole patterns has little effect on extremum locations. However, as for the demodulation algorithm abstracting the cavity length from extremum locations, one major challenge is that extremum locations are periodically distributed, so if we only focus on the phase change, we can’t distinguish the extreme points, that is, we can’t track a certain order of low coherence interference fringes. To achieve this goal, in this paper, extremum locations adjacent to the envelope center are selected as the target for tracking. According to Equations (4) and (5), the locations of extreme value and the envelope center satisfy the following linear relationship:

(6) $$h_{m}=\left(\left(\Delta n_{0}+\beta k_{0}\right)/\Delta n_{0}\right)h_{c}+\left(1/\Delta n_{0}\right)m\pi /k_{0}$$

As the cavity length varies, the slope of the extremum locations and envelope center trends should be

(7) $$\begin{array}{l} \frac{\delta h_{m}}{\delta d}=2/\Delta n_{0}\\ \frac{\delta h_{c}}{\delta d}=2/\left(\Delta n_{0}+\beta k_{0}\right) \end{array}$$

As $\beta k_{0}\ll \Delta n_{0}$ in birefringent crystal wedges, Equation (7) illustrates that the changes of envelope center and extremum locations are approximately the same, this means that envelope center and extremum locations always remain relatively static within the measurement range, which enables envelope center to serve as a marker for extremum locations determination. To increase the tolerance of envelope extraction deviation, we first predict the extremum locations in the neighborhood by the envelope center, and then accurately search the extrema with the predicted locations as the reference. To suppress the single extremum position error caused by shot noise, the middle location of the pair of peak/valley closest to the envelope center is utilized to recover the cavity length.

Based on the above analysis, the principal steps of the proposed method are as follows:

1. The calibration of the demodulation model
  1.1 set a series of sample points at equal intervals within the measurement range, and monitor the variation curves of envelope center.
  1.2 track the target extremum locations in the vicinity of envelope center, and linear -fit the variation trends of the peak and valley closest to the envelope center.
  1.3 track the trend of peak/valley locations and calculate the mean value to get the curve of middle location.
2. The demodulation of a given F-P sensor
  2.1 extract the envelope of the PLCI pattern, and predict the positions of the closest peak and valley according to the calibration trend.
  2.2 center on the predicted peak/valley locations, and search for the extreme points in their neighborhood.
  2.3 take the searched extremums as the peak/valley locations, calculate the middle location, and derive the cavity length.

For the proposed demodulation method, the resolution and accuracy of the whole system is mainly determined by the tracking precision of the envelope center, extremum locations, and middle location. The next section will focus on the high precision data processing algorithm, wherein the wavelet tools including WTD and MWEE are employed to extract the sensing information from the interferometric pattern.

## 3. PLCI Fringe Wavelet Processing

The processing algorithm utilized in this paper contains three portions: denoising based on WTD, envelop extraction based on MWEE and extremum locations tracking.

### 3.1. WTD

**Figure 3:**
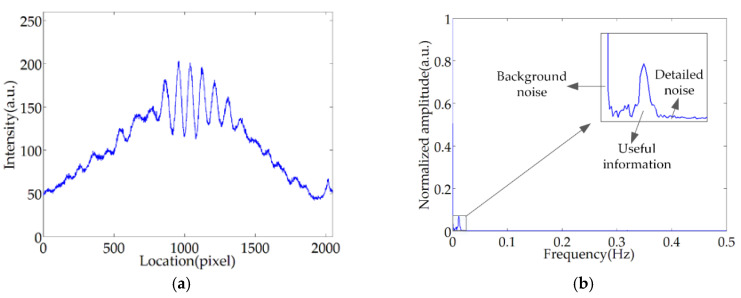
Low-coherence interference fringe: (**a**) spatial distribution of the fringe; (**b**) frequency domain information of the fringe.

Because of the light source fluctuations, electrical noise, etc., the actual interferogram is different from the ideal case shown in Figure 2c. To analyze this degeneration, we employed the experimental setup shown in Figure 2a to gather actual images of PLCI patterns, and recorded the fringe locations and the light intensity as pixels (ranging from 1 to 2048) and grayscale values (ranging from 0 to 255), respectively. As shown in Figure 3a, an actual pattern involves background noise, glitches, and amplitude distortion. Through Fourier transform, the amplitude-versus-frequency curve of the pattern is shown in Figure 3b, an evident overlap between the useful information and the noise could be observed, which makes it difficult to completely separate the signal from the noise in the frequency domain.

The WTD method is used to filter out detailed noise, such as electrical noise. The denoising method utilized in this paper includes three parts: the wavelet decomposition, the coefficient quantization, and the wavelet reconstruction [23,24]. The lower layers of the wavelet decomposition coefficients correspond to the detailed components of the signal, and the higher layers correspond to the approximate components, so the useful fringe and the noise can be separated. The threshold function is used to process the wavelet coefficients, which can weaken the wavelet coefficients corresponding to the detailed noise. After that, the wavelet coefficients are reconstructed to obtain a denoised fringe. 

The selection of wavelet basis function, number of decomposition layer, threshold value, and threshold function are all critical factors affecting the denoising effect. The heursure threshold standard and soft threshold function are selected, and the threshold is adjusted according to the estimation of the first-layer decomposition noise level. In this way, the noise can be eliminated, and additional vibrations can be avoided. In this paper, the effects of several standard wavelet basis functions, different wavelet orders, and different decomposition layers on the denoising effect have been studied.

**Figure 4:**
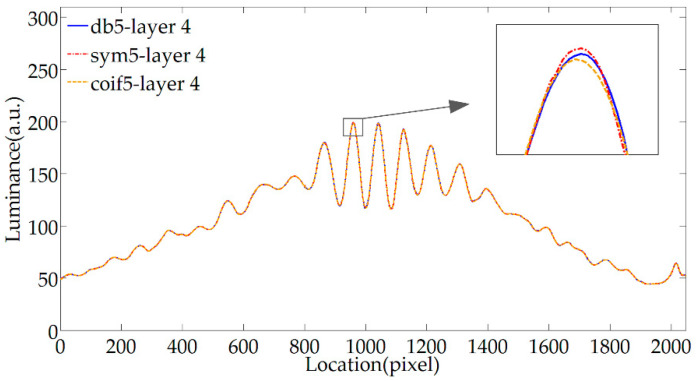
Filtering effects of different wavelet basis functions.

Figure 4 shows the denoising effect of the db5, sym5, and coif5 wavelet basis function. The three common wavelet basis functions belong to different wavelet families Daubechies (dbN), Symlets (symN), and Coiflets (coifN), respectively. The wavelet order is the same, and they all have continuity and orthogonality. The results show that these wavelet basis functions have visible denoising effects, but there are differences in local locations. The sym5 wavelet is consistent with the db5 wavelet in terms of support length, filter length, and vanishing moment order. The vanishing moment reflects the flatness of the filter and determines the ability to approximate the smooth signal. The difference is that the sym5 wavelet has better symmetry, which can reduce the phase distortion when the signal is decomposed and reconstructed. After testing, the detailed noise is concentrated in the coefficients below the number of decomposition layers 4, so the number of decomposition layers is selected to be 4. It can be seen from Figure 4 that when the number of decomposition layers is determined, the smoothness of the fringe is close after denoising with the db5 wavelet and the sym5 wavelet, but the symmetry of the fringe processed by the sym5 wavelet is better, so the fringe phase information is more accurate after denoising. The accuracy of the demodulation is directly related to the accuracy of the judgment of the extremum locations, so the phase distortion has a great influence on the accuracy of the demodulation. Therefore, the sym5 wavelet has advantages over the db5 wavelet. The coif5 wavelet is approximately symmetrical as the sym5 wavelet, but the vanishing moment of the former is 10, and the latter has a vanishing moment of 5, so the coif5 wavelet has stronger denoising and smoothing capabilities. As can be seen from Figure 4, the signal phase after the coif5 and sym5 wavelet denoising is close, but the former has better local smoothness because the vanishing moment is twice that of the latter. Therefore, when the coif5 wavelet is used to denoise a specific signal, the choice of parameters is more. In summary, the Coiflets wavelet can avoid phase distortion and has a strong smoothing ability, so the Coiflets wavelet is selected for denoising processing.

**Figure 5:**
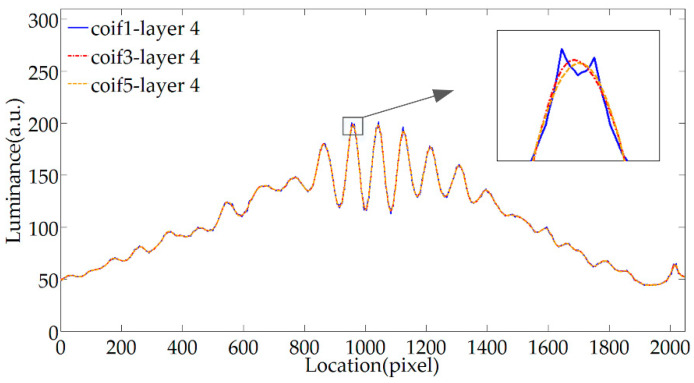
Filtering effect of different wavelet orders.

Figure 5 shows the denoising effect of the coif1, coif3, and coif5 wavelet. They all belong to the coifN wavelet family, and the wavelet order N is different. The characteristic of the coifN wavelet is that as the order N increases, the higher the vanishing moment, the better the smoothness of the denoising signal. It can be seen from Figure 5 that when the number of decomposition layers is the same, the smoothing ability of coif5 wavelet is relatively strong, and the higher the wavelet order, the more parameters are available for the decomposition layer. In summary, the coif5 wavelet with high wavelet order is selected for denoising processing.

**Figure 6:**
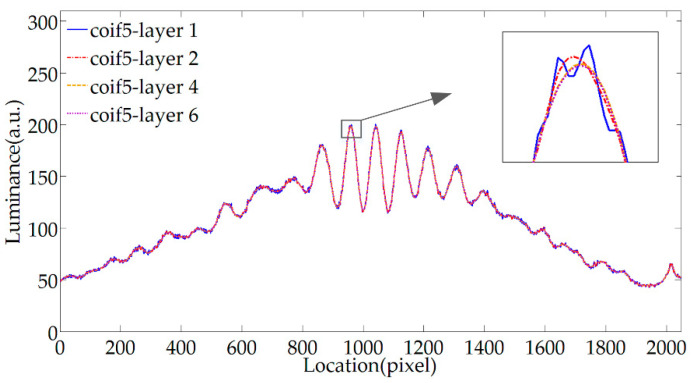
Filtering effect of different decomposition layers.

The fringe signal is denoised with 1, 2, 4, and 6 decomposition layer, the effect is shown in Figure 6. The different decomposition layers of wavelet transform correspond to the information of different scales of the signal, the low layer corresponds to the noise, and the high layer corresponds to the outline part of the fringes. The detailed noise of the fringes is caused by electrical noise and other factors. The scale and signal-to-noise ratio of the noise contained in the actual fringes are unknown, so it is impossible to directly determine the parameters of the decomposition layer. However, the feature of scale analysis makes the ideal number of denoising layers exist in wavelet denoising, which effectively filters out noise and ensures minimum signal feature distortion. It can be seen from Figure 6 that the higher the number of decomposition layers, the smoother the result, when the number of decomposition layers reaches 4, the detailed noise has been well removed. If the number of decomposition layers exceeds the appropriate value, the improvement of the noise suppression effect is no longer evident, and it may affect the shape of the valid signal. In summary, the number of wavelet decomposition layer is selected as 4 when denoising the stripes.

The above content analyzes the influence of the relevant parameters of wavelet denoising on the denoising effect using an actually collected fringe. Finally, the coif4 wavelet is selected, and the decomposition layer four is used to effectively filter out the detailed noise and extract the effective fringe information, which retains the signal characteristics.

### 3.2. MWEE

Complex Morlet wavelet is mostly used for signal feature extraction [25,26,27]. Its mathematical definition can be expressed as

(8) $$\psi_{a,b}\left(x\right)=\frac{1}{\sqrt{\pi f_{b}a}}\exp\left(\frac{-{\left(x-b\right)}^{2}}{2f_{b}a^{2}}\right)\exp\left(2j\pi f_{c}\frac{x-b}{a}\right)$$

where $f_{b}$ is the bandwidth parameter, $f_{c}$ is the center frequency, a is the wavelet scale parameter, and b is the translation factor. The wavelet basis function is composed of the real part and the imaginary part, the amplitude-frequency characteristics are the same, and the phase difference is 90°. The wavelet basis function is consistent with the form of PLCI fringe, which is a single frequency carrier with Gaussian envelope modulation, the result of wavelet transform processing on the fringe is

(9) $$W_{I}\left(a,b\right)=\langle I(x),\psi_{a,b}\left(x\right)\rangle =W_{Ir}\left(a,b\right)+jW_{Ii}\left(a,b\right)$$

where I(x) is the fringe distribution, $W_{Ir}\left(a,b\right)$ is the real part of the wavelet coefficient, and $W_{Ii}\left(a,b\right)$ is the imaginary part of the wavelet coefficient. The real part and imaginary part of the coefficient are interpolations to the envelope and have the orthogonal characteristic so that the envelope can be expressed as the coefficient mold:

(10) $$I_{e}(x)=\sqrt{W_{Ir}^{2}\left(a,b\right)+W_{Ii}^{2}\left(a,b\right)}$$

When performing a one-dimensional continuous wavelet transform on the fringe, set the bandwidth parameter $f_{b}$, the center frequency $f_{c}$, and the scale parameter a of the transformation, where $f_{b}$ and $f_{c}$ are parameters related to the wavelet basis function, and the function is expressed as cmor $f_{b}-f_{c}$. These two parameters affect the initial shape of the wavelet basis function. Based on the wavelet denoising process, the influence of each parameter on the effect of envelope extraction has been studied.

**Figure 7:**
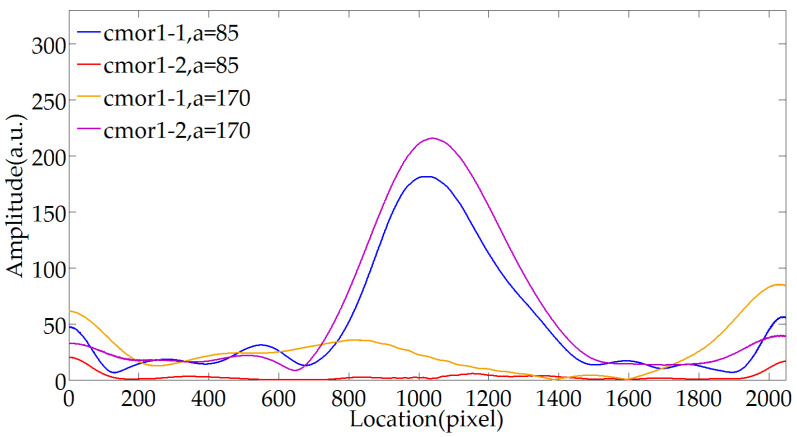
Influence of the center frequency and the scale parameter on envelope extraction.

Figure 7 shows the effect of the center frequency $f_{c}$ and the scale parameter a on the envelope extraction results. When transforming a signal using complex Morlet wavelets, the wavelet coefficients reflect the projection of the signal in the direction of the wavelet at this scale, so when the wavelet waveform is more similar to the original signal, the more significant the projection amount, the effective information can be more prominent. The center frequency $f_{c}$ corresponds to the initial scale of the wavelet basis; the scale parameter a indicates that the wavelet basis is stretched by a corresponding multiple, and the wavelet transform is performed on the fringe signal using the stretched wavelets of different scales to obtain the corresponding wavelet coefficients. Therefore, when complex Morlet wavelet is used to extract the envelope, the center frequency and scale parameter jointly affect the extraction effect. From Equation (5), the period of the PLCI fringe with respect to the thickness of the optical wedge satisfies:

(11) $$T_{h}=\frac{2\pi }{k_{0}\Delta n_{0}}$$

Further projected on the LSC, the cycle of acquiring fringe is

(12) $$T_{L}=\frac{2\pi }{k_{0}\Delta n_{0}\tan\gamma l_{p}}$$

where γ is the wedge angle, and $l_{p}$ is the size of a pixel. It can be seen that the fringe period is only related to the center wavenumber of the light source and the optical wedge parameters. The analysis of the collected fringes shows that the period is always about 85 pixels. Therefore, when demodulating different cavity lengths, the wavelet transform parameters need not be replaced. To obtain the effect of the center frequency $f_{b}$ and the scale parameter a on envelope extraction, set the bandwidth parameter unchanged, and the envelope is extracted at the scale parameter of 85 and 170 using cmor1-1 and cmor1-2, respectively. The results are shown in Figure 7, indicating that when $a/f_{b}=85$ is satisfied, the envelope can be better extracted. In summary, when the scale of the wavelet function after stretching is close to the fringe interval, a better processing effect can be achieved.

**Figure 8:**
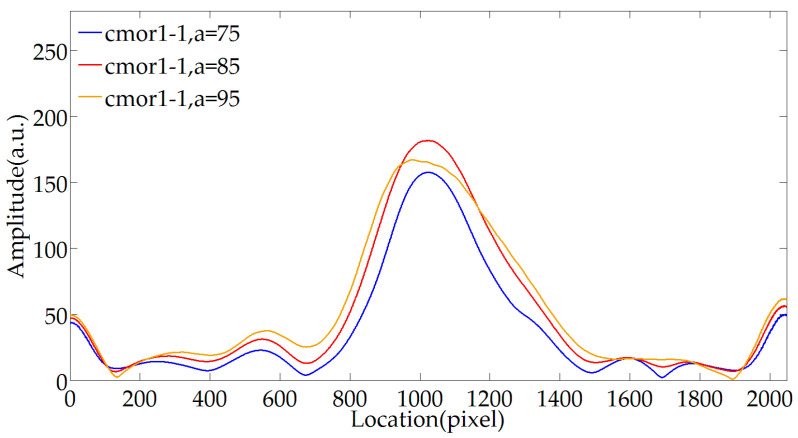
Impact of bandwidth parameter on envelope extraction.

Figure 8 shows the results of further exploration of the effect of the wavelet scale on envelope extraction. Use cmor1-1 wavelet for wavelet transformation, and the scale parameters are set to 75, 85, and 95, respectively. The wavelet scale changes around the size of the fringe period. The results show that the closer the wavelet scale is to the fringe period, the better the envelope extraction effect. When it is slightly smaller than the period, the coefficient amplitude is attenuated, but the envelope profile can be obtained. When it is slightly larger than the period, the envelope is distorted, and the distortion is due to the background noise caused by the spatial distribution of the light emitted from the fiber. Therefore, when using the wavelet tool to obtain the envelope center, there is an individual tolerance in the selection of the scale parameter, but it should not be higher than the optimal scale parameter value. In this paper, the center frequency $f_{c}$ is selected as 1, and the scale parameter a is selected as 85.

**Figure 9:**
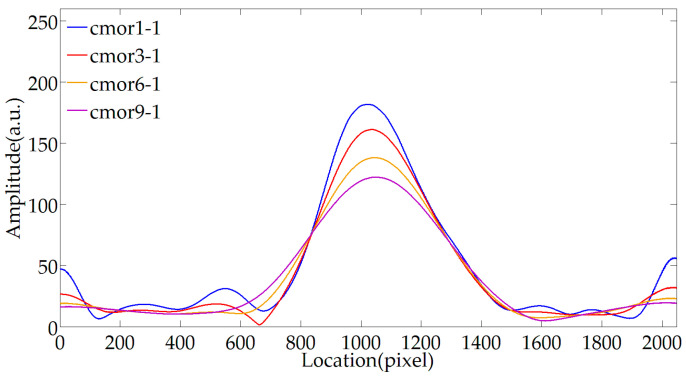
Impact of bandwidth parameter on envelope extraction.

Figure 9 shows the effect of bandwidth parameter $f_{b}$ on envelope extraction. The bandwidth parameter corresponds to the time domain width of the wavelet basis function. The larger the bandwidth parameter, the longer the support length. When the scale parameter a is 85, and the center frequency $f_{c}$ is 1, the bandwidth parameter $f_{b}$ is changed. The fringe envelope is extracted by cmor1-1, cmor3-1, cmor6-1, and cmor9-1. The results show that the larger the bandwidth parameter, the more wavelet coefficients with high amplitude, the wider the curve, the better the symmetry. However, the envelope center of the gentler envelope is more difficult to determine. The number of distinguishable cycles of PLCI stripe is about 6, so when the bandwidth parameter is selected as 6, the wavelet and fringe forms are closer, and a better processing effect can be achieved.

The following compares and analyzes the difference between the complex Morlet wavelet method and the Fourier transform method to extract the fringe envelope and compares the processing of the original signal and the denoised signal, respectively. It can be known from the theory that the principle of the two envelope extraction methods is to construct two orthogonal signals interpolating the envelope, so the square sum calculation can be used to obtain the envelope. The wavelet transform can overcome the influence of noise to a certain extent due to the decorrelation, but the Fourier transform method is more dependent on the denoising process.

**Figure 10:**
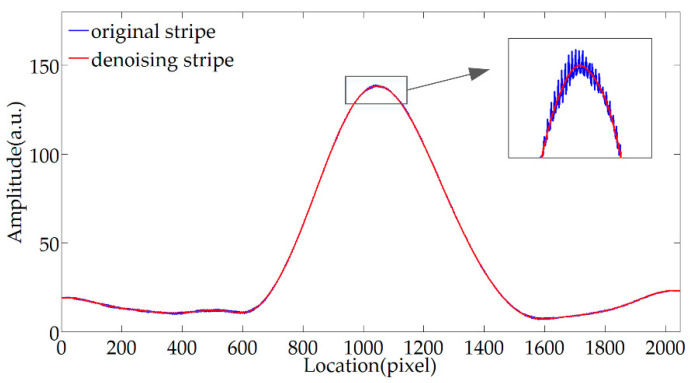
Complex Morlet envelope extraction effect before and after wavelet threshold denoising (WTD).

Figure 10 shows the results of complex Morlet envelope extraction processing on the original fringe and the fringe after WTD processing. It can be seen that the envelope extraction on the original stripe directly can overcome the effect of detail noise to a certain extent, but there are still slight glitches. The stripe envelope extraction after the denoising process can eliminate glitches and has excellent smoothness. Therefore, WTD plays an essential role in stripe information extraction. At the same time, it can be seen that when the complex Morlet wavelet is used to extract the envelope, there may be a stretch transform in amplitude, but the information about the envelope center and the extremum locations required by the demodulation theory are only related to the phase information, so there is no need to consider the proportional change in amplitude. The following uses the normalized envelope to compare the effect of complex Morlet wavelet and Fourier transform in envelope extraction.

**Figure 11:**
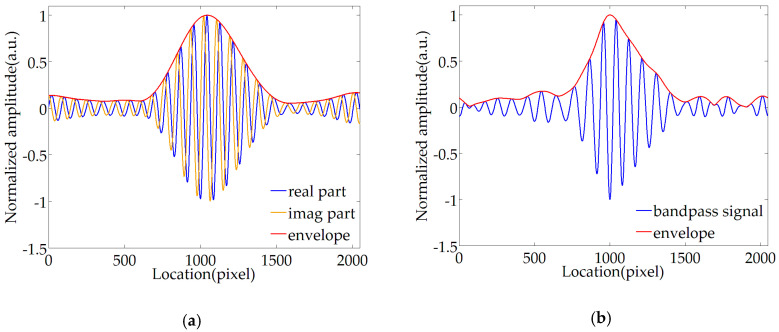

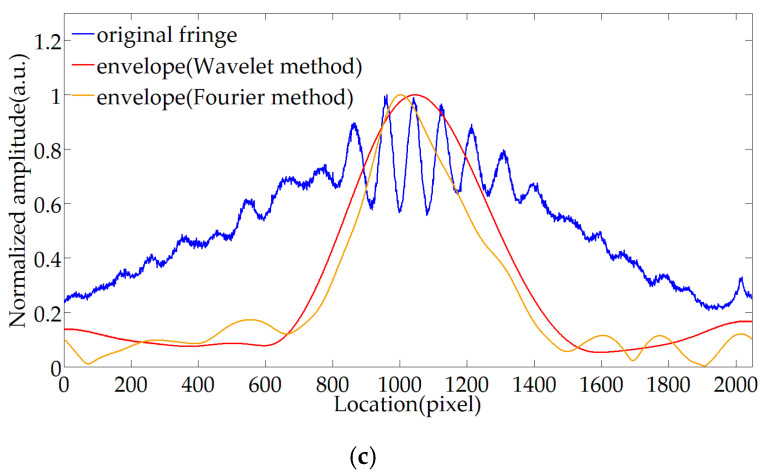
Comparison of envelope extraction methods: (**a**) wavelet normalized envelope extraction effect; (**b**) Fourier normalized envelope extraction effect; (**c**) comparison between envelope curve and original fringe.

Figure 11a shows the real part, the imaginary part of the normalized wavelet transform coefficients, and the envelope obtained by modulo operation. Due to the non-correlation between background noise and wavelet function, the background noise can be eliminated when extracting the envelope, and the useful components of information are highlighted. Furthermore, this method effectively overcomes the influence of distortion and can restore the Gaussian envelope. At the same time, the real part of the wavelet transform coefficient is consistent with the form and phase of the fringe.

Figure 11b shows that the effect of extracting the envelope using the Fourier transform envelope extraction method based on band-pass filtering, but the frequency domain filtering may affect the useful components. The smoothness of the envelope is weak, and even multiple peaks may occur, affecting the accuracy of the envelope center.

Taking the original fringe as a reference, the envelope extracted by wavelet method and Fourier transform method is compared, as shown in Figure 11c. The comparison results show the effect of MWEE is significantly better than the traditional Fourier transform envelope extraction method. This new method has excellent flexibility and smoothness, can eliminate the distortion of the envelope, highlight useful information, and avoid background noise interference.

### 3.3. Extremum Locations Tracking

The above content realizes the denoising and envelope extraction of PLCI fringes and completes the prerequisites for demodulation. The extremum locations are predicted from the calibrated linear relationships between the extremum locations and the envelope center. Moreover, with the predicted locations as the center, the real locations of the peak and valley value are searched within a range of the fringe interval.

**Figure 12:**
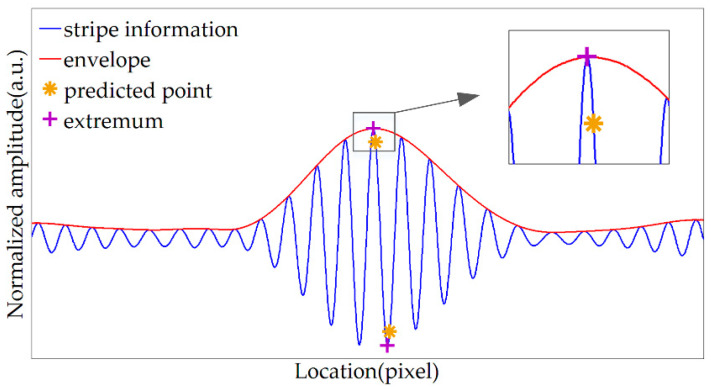
Determine the extremum locations of the target order fringe.

When using the envelope center to predict the extremum locations of specified order, due to the complexity of the influencing factors of the amplitude, the envelope center is prone to deviation, so the predicted locations generally deviate from the actual extremum locations. However, the predicted position in this paper is only used for rough positioning and is used to restrict the search range when searching for actual extremum locations. Therefore, there is a certain tolerance for the deviation of the envelope. Therefore, this method can avoid jumping errors and is more practical. As shown in Figure 12, the locations predicted by the envelope center are near the actual extremum locations, and the algorithm can successfully search for the peak location and bottom location of the target order fringe.

## 4. Experimental Results and Discussions

**Figure 13:**
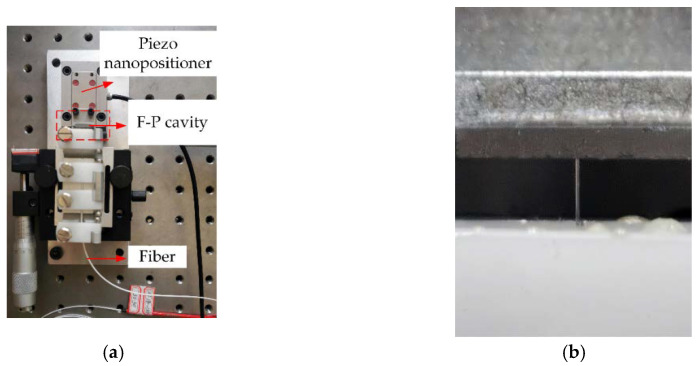
F-P cavity structure: (**a**) F-P cavity system composed of optical fiber and quartz glass slide; (**b**) Local structure of F-P cavity.

The central wavelength of the GLS is 650 nm, and the full width at half maximum is 75 nm. T The F-P cavity environment consists of an optical fiber end face and a reflective glass installed on a piezo nanopositioner, as shown in Figure 13. Precise adjustment of cavity length can be achieved by controlling the displacement of the piezo nanopositioner to simulate the working process of F-P sensors, the closed-loop travel is 12 μm, the displacement resolution is 0.05 nm, and the closed-loop linearity error is 0.03%. The material of the selected birefringent crystal optical wedge is MgF2, the wedge angle is 5.5 ± 0.10°, the finish of the wedge is 20/10 and the orientation accuracy is less than 6 Arc min. The birefringence of the birefringent crystal wedge approximately satisfies the following formula: $\Delta n=0.011766+3.2\times 10^{-5}\left(k-9.67\right)$. The LA-CM-02K-08A line scan camera (Teledyne DALSA, Canada) that collects stripe has a resolution of 2048 × 1, a pixel bit depth of 8/12 bits, and a pixel size of 7.04 × 7.04 µm.

Calculated by Equation (12), the number of pixels corresponding to the period of the fringes is about 81.5, and the actual value is 85. The reason for the deviation is the non-perpendicular incidence of light or the angle of refraction of the optical wedge. The effect of light tilt is equivalent to widening the stripes and does not affect the distribution of the stripes. Before demodulation, the demodulation system needs to be calibrated.

### 4.1. Calibration Work and Process Analysis

**Figure 14:**
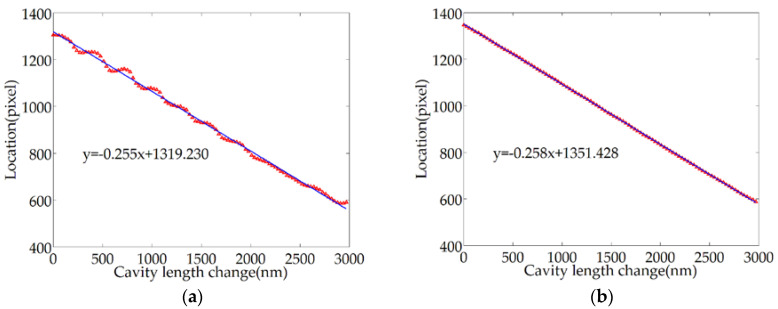
Characteristic curves of sample points: (**a**) the curve of envelope center-cavity length obtained by wavelet processing; (**b**) the curve of middle location-cavity length obtained by the algorithm in this paper.

The cavity length was controlled to vary within a range of 3 μm with a 30 nm step distance, and the interferograms of the 100 sample points were acquired. Processed by the wavelet tool described in Section 3, the variation curve of envelope center is shown in Figure 14a, the nonlinear error of this curve is around 3.79%, which is mainly contributed by the amplitude distortion of GLS. Tracking a fixed order of fringes in the vicinity of the envelope center, the relationship between the peak location $h_{pt}$ and the envelope center $h_{e}$ is approximately satisfied:

(13) $$h_{pt}=1.014h_{e}-8.128$$

The relationship between the valley location of this order $h_{vt}$ and the envelope center $h_{e}$ is approximately satisfied:

(14) $$h_{vt}=1.014h_{e}+35.349$$

The coefficient is 1.014, which is slightly higher than 1. The reason is that the relative deviation of the envelope and fringe caused by the dispersion of the birefringent crystals, which verifies the correctness of Equation (6).

For the length of the cavity to be measured, the extremum locations can be predicted by the envelope center through the above relationships. Under special circumstances, if the deviation between the predicted extremum locations and the actual locations exceeds half a cycle due to the envelope deviation $\delta_{h_{c}}>\pi /\left(k_{0}(\Delta n_{0}+\beta k_{0})\right)$, the algorithm based on this algorithm will be invalid due to the jump error. According to the above two fitting formulas, the maximum deviation of the predicted location caused by the nonlinear error of the envelope center is about 29.4 pixels, and the fringe cycle is approximately 85 pixels, so the prediction deviation is allowed to be 42.5 pixels. Therefore, the extremum locations falls within the search range of a fringe interval with the predicted locations as the center, and there is no step jump error across the entire range, so the extremum locations of the target order fringe can be accurately searched. The curve obtained by searching the target order middle location using the algorithm proposed in this paper is shown in Figure 14b. It can be seen that the middle location can be accurately positioned in the entire scanning range, and the change curve of the middle location of the target order is shown in Figure 14b. The accuracy of demodulation using the extremum locations of the fringe is significantly higher than that of using the envelope center.

For the same group of images, the centroid method and the Fourier transform method are used to obtain the envelope center, respectively. The results are compared with the effects of the algorithm in this paper, and related discussions are conducted.

**Figure 15:**
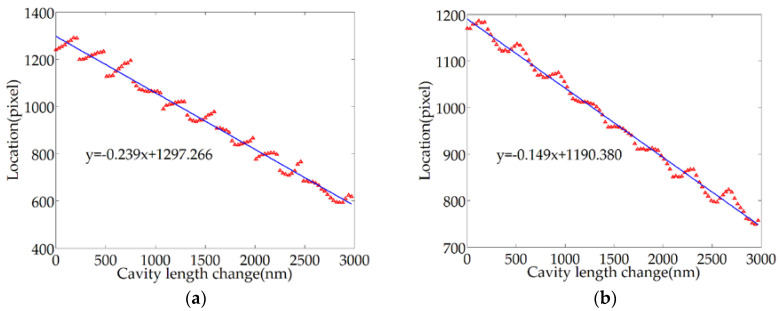
The curve of the envelope center: (**a**) the curve of envelope center-cavity length obtained by centroid method; (**b**) the curve of envelope center-cavity length obtained by Fourier transform.

Both the centroid method and the Fourier transform method need to denoise the stripes. Within the range of cavity length scanning, the envelope center curves obtained by the two methods are shown in Figure 15a,b, respectively. Both curves have large fluctuations, and the nonlinear errors are 6.90% and 10.87%, respectively. The maximum deviation of the predicted location caused by the nonlinear error of the envelope center is about 53.5 pixels and 84.3 pixels, which are greater than 42.5. Therefore, it is easy to happen that there is no extremum or even multiple extremums in the search interval, and at the same time, the order jump error cannot be avoided. Because of the distortion of the envelope, the average effect of the centroid method makes its envelope linearity higher than the Fourier transform method. In summary, the data processing method of wavelet transform assists the extremum search algorithm can effectively track the extremum of a fixed order, and the demodulation scheme has better error tolerance.

### 4.2. Repeatability Accuracy Test

**Figure 16:**
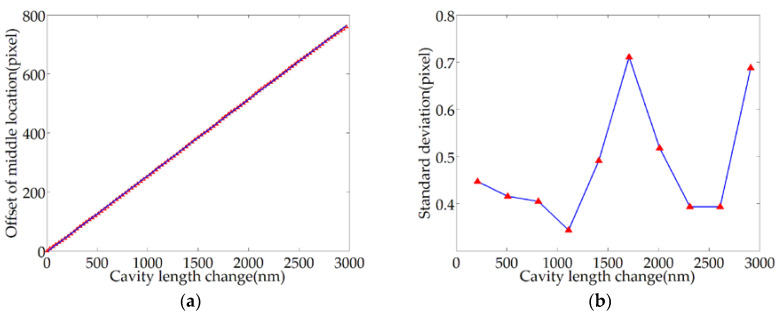
Test results for repeatability: (**a**) relative offset curve of the middle location; (**b**) distribution of standard deviation representing sample points.

Within the variation range of the cavity length of 3 μm, 100 sample points were uniformly set at a distance of 30 nm, the relative deviation of the middle location was recorded, and the measurement was repeated 11 times. The relative offset curve of the middle location of the target order extrema is shown in Figure 16a. The standard deviations of the sample points calculated by the Bessel formula are shown in Table 1. Ten of the sample points are selected to draw the error bar chart, as shown in Figure 16b.

The repeatability standard deviation is less than 0.71 pixels, which corresponds to a deviation of 2.71 nm for cavity length. Therefore, the repeatability of the cavity length is better than $U=t_{\left(10,0.05\right)}\times S=2.228\times 2.71\mathrm{nm}\approx 6.04\mathrm{nm}$ (the confidence probability is 95%). The repeatability is about 0.21% of the whole range. The test results show that the repeatability standard deviation is less than the one-pixel value because 11 groups of test samples correspond to the same series of the cavity length. In most cases, the middle location of extrema can be located in the same pixel position at the same sampling point. Therefore, it is reasonable that the repeatability standard deviation is less than one-pixel value. Moreover, the accuracy can be improved by reducing the inclination of the BW and improving the acquisition device.

### 4.3. Resolution Test

The resolution of the proposed method is evaluated by a series of step tests. In each test, PZT was set to move with a specified interval per 5 s, and ten images at each location were recorded by the proposed demodulation system, which would be shaped like steps if the method could resolve the changes of the cavity length of the F-P sensor.

**Figure 17:**
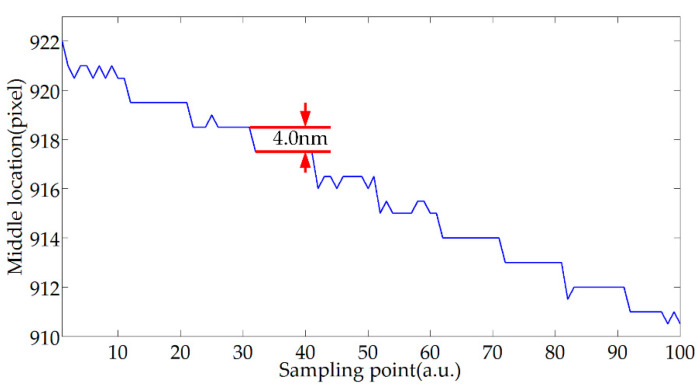
Test results for resolution.

To find the critical resolution, this paper gradually decreased the intervals from 6.0 nm to 2.0 nm, as shown in Figure 17, the resolution of the proposed method is better than 4.0 nm. After analysis, the fringe offset corresponding to this resolution is close to the size of a single pixel, so higher resolution can be achieved by improving the collection device.

The above experimental results show that the designed demodulation algorithm scheme is feasible, has a strong tolerance for the spectral deviation of the light source, and has high demodulation accuracy and resolution. At the same time, the wavelet tool is suitable for extracting the useful information of the fringes, which can completely denoise and obtain an accurate envelope center.

## 5. Conclusions

To improve the accuracy of F-P sensor demodulation, a novel PLCI fringe processing scheme is proposed. The wavelet tools are used to extract the useful information of the stripes, and the envelope center is used to locate the middle location of a fixed-order stripe for demodulation. The stripe information extraction process includes coif WTD processing, MWEE, and search of stripe extremum locations of a fixed order. Calculate the middle location and the cavity length according to the calibration curve of the location relative to the cavity length. The effectiveness of this scheme is verified by using optical fiber and a glass sheet to form an air F-P cavity. The experimental results show that the wavelet tool can effectively overcome the influence of noise and extract the envelope well. The search method of the extremum locations can avoid jump errors, and the demodulation scheme can accurately demodulate the cavity length. Within the range of 3 μm cavity length, the repeatability accuracy of demodulation reaches 6.04 nm, and the resolution reaches 4.0 nm. Before this, the limiting factors for demodulation accuracy were the spectral deviation of the light source and the noise level. This scheme converts the limiting factors into the inclination of the BW, so the accuracy can be further improved by improving the demodulation device.

At present, we built a principle prototype during the test but did not carry out the field experiment. In the demodulation system, the light source spectrum and wedge birefringence characteristics change with the environment and time. Therefore, the light path can be solidified, the light source spectrum and the fringe distribution corresponding to the wedge birefringence characteristics can be calibrated regularly to realize field measurement.

## Figures and Tables

**Table 1:** Standard deviation (Std) of sample points.

Point	Std	Point	Std	Point	Std	Point	Std	Point	Std
1	0.00	21	0.55	41	0.55	61	0.38	81	0.38
2	0.38	22	0.39	42	0.55	62	0.39	82	0.39
3	0.55	23	0.47	43	0.40	63	0.32	83	0.32
4	0.51	24	0.34	44	0.44	44	0.44	84	0.44
5	0.42	25	0.32	45	0.44	65	0.45	85	0.45
6	0.56	26	0.47	46	0.47	66	0.51	86	0.51
7	0.52	27	0.34	47	0.50	67	0.32	87	0.32
8	0.45	28	0.40	48	0.49	68	0.42	88	0.42
9	0.56	29	0.39	49	0.42	69	0.52	89	0.52
10	0.39	30	0.32	50	0.23	70	0.34	90	0.34
11	0.25	31	0.47	51	0.46	71	0.56	91	0.56
12	0.39	32	0.39	52	0.35	72	0.45	92	0.45
13	0.34	33	0.47	53	0.34	73	0.42	93	0.42
14	0.34	34	0.56	54	0.40	74	0.51	94	0.51
15	0.52	35	0.34	55	0.41	75	0.39	95	0.39
16	0.52	36	0.45	56	0.25	76	0.40	96	0.40
17	0.42	37	0.39	57	0.60	77	0.63	97	0.63
18	0.42	38	0.34	58	0.71	78	0.42	98	0.42
19	0.42	39	0.34	59	0.47	79	0.39	99	0.39
20	0.26	40	0.38	60	0.38	80	0.45	100	0.45
